# Is U.S. health care an appropriate system? A strategic perspective from systems science

**DOI:** 10.1186/1478-4505-7-1

**Published:** 2009-01-02

**Authors:** Ivo P Janecka

**Affiliations:** 1 Health Research International, 333 Westbrook Rd, St Helena Island, SC 29920, USA

## Abstract

**Context:**

Systems science provides organizational principles supported by biologic findings that can be applied to any organization; any incongruence indicates an incomplete or an already failing system. U.S. health care is commonly referred to as a system that consumes an ever- increasing percentage of the gross domestic product and delivers seemingly diminishing value.

**Objective:**

To perform a comparative study of U.S. health care with the principles of systems science and, if feasible, propose solutions.

**Design:**

General systems theory provides the theoretical foundation for this observational research.

**Main Outcome Measures:**

A degree of compliance of U.S. health care with systems principles and its space-time functional location within the dynamic systems model.

**Results of comparative analysis:**

U.S. health care is an incomplete system further threatened by the fact that it functions in the zone of chaos within the dynamic systems model.

**Conclusion:**

Complying with systems science principles and the congruence of pertinent cycles, U.S. health care would likely dramatically improve its value creation for all of society as well as its resiliency and long-term sustainability.

Immediate corrective steps could be taken: Prioritize and incentivize *health *over *care*; restore fiscal soundness by combining health and life insurance for the benefit of the insured and the payer; rebalance horizontal/providers and vertical/government hierarchies.

## Background

U.S. health care is commonly referred to as a system that consumes an ever-increasing percentage of the gross domestic product while delivering seemingly diminishing value (seen as a relationship among quality, risk, and cost) and continuously eluding remedies. [1-3] Such observations point to chronic fundamental systemic organizational misalignments. The purpose of this study was to examine the degree of compliance of U.S. health care with systems science principles, which approximate the framework of successful organizational settings found in biology; if followed, these guidelines should optimize societal health care.

The current state of health care represents a structural and functional outcome of its historic contradictions with systems science principles, that is, a mismatch of vertical (those that represent government legislature) and horizontal (patients with providers involved in health care) hierarchies and the failure of components relationships, all resulting in substantial damage to the overall system's self-organizing capacity. In view of the changing dominant economic and political cycles, health care is visibly failing and showing signs and symptoms that indicate that its survival is at risk (e.g., diminishing value, runaway cost, breakdown of relationships, demoralized providers, etc.). [4,5] (Additional file 1, Part 1). A search for an alternate perspective leading to a comprehensive solution seems paramount. Einstein's comment that "no problem can be solved from the same consciousness that created it" encourages this approach. [6]

Healthcare is encountering major challenges on key fronts when identified through systems science:

Components: not all of society is included; most participating subsystems (i.e., providers/suppliers) do not optimize the "whole," but maximize the "self" instead.

Relationships: exist but without solid, course-adjusting feedback loops; a disconnect exists among hierarchies, and fiscal/legislative control is not systems derived; information transfer is not uniform and is heavily compartmentalized; complexity exists primarily in its disorganized form, expressing strong prevalence of chaos and a high degree of entropy.

Value: not consistently created

Boundary: not functionally semipermeable.

Based on the aforementioned observations, analyzed from scientific and public domain publications dated within the last decade, the following research questions were asked:

1. Is U.S. health care a system, as defined by systems science?

2. What is the state of its functioning within the dynamic systems model?

## Concepts

A system consists of a large number of variable components engaged in ongoing relationships that are three-dimensional and multidirectional, fully dependent on feedback loops and the utilization of common information transfer pathways. A system has a boundary that delineates and protects the internal environment from the external environment but also facilitates dynamic system adaptation and mutual impact; these two functions are inseparable. The boundary's dynamic properties are analogous to biologic semipermeability describing a selective exchange. (Additional file 1, Parts 1, 2 and 11).

A system, regardless how large, is an integral part of an even larger system and likewise contains multiple, smaller interrelated systems, known in this context as subsystems. Within space-time, systems progress through cycles. (Additional file 1, Part 9) A complex adaptive system is an open system large enough to be capable of ongoing favorable adaptation of its internal function and structure to the external environment while respecting systems science principles. The best example of a complex adaptive system that epitomizes systems science principles and their interrelationships with cycles in function and structure is the human body. Its evolutionary adaptation has been the result of ongoing system-wide modulation to external demands and internal capabilities. System-compliant cycles/oscillations express self-similarity and self-affinity. Depending upon a system's temporal location within a dominant larger cycle, the outcome of a system's reactivity may vary dramatically. This realization is critical to our understanding of why, for example, identical healthcare interventions or legislated policies may lead to vastly different outcomes among similar clients. For example, decisions made in congruence with a dominant cycle will likely result in positive and magnified outcomes, whereas those decisions that are incongruent or not in synch with the evolving cycle will likely have poor or detrimental outcomes. The recently developed dynamic systems model graphically demonstrates the expected variability and predictability of functional outcomes depending upon a system's temporal location, symbolized as a pendulum, within this model. [7] If a system is functioning within the health territory, namely, its steady state of ongoing adaptation, it exhibits a high degree of dependability, resiliency, and evolvability. On the other hand, if a system is functioning outside of this territory, that is, in the zone of chaos or growing entropy, it is failing.

Relationships in a system are not static; they reflect the dynamic process of a system's functioning and precede the formation of a system's structure. The types of relationships correspond to the phases of a system's space-time location within the dynamic systems model. The evolution of systems into stable, pendulum-like oscillations requires system-enhancing attractors that facilitate self-organization. [8] Capra pointed out that the development of an organism, for example, is characterized by a series of bifurcations corresponding to different attractors and that at the edge of chaos, the number of attractors in such a network is approximately equal to the square root of the number of its elements/components. [9] In a social system, by comparison, any person or even an idea may emerge as an attractor, creating new patterns of information and self-organizing emergence. [10] According to Wheatley, although numerous attractors play roles of different intensity, meaning is the most potent. [6]

General systems theory, originally supported by biologic findings applicable to organizations, provided the theoretical foundation for this study; additional principle insights came from Ashby, Bertalanffy, Boulding, Capra, Hatch, and Wheatley. [6,11-15] A recently described dynamic systems model also has been used to evaluate the key functional characteristics of health care and gage their compliance with systems science and cycles. [7]

Bertalanffy believed in the interdisciplinary applicability of systems science resulting from the existence of "isomorphism, [a] parallelism of general cognitive [and structural] principles in different fields" (p. xviii). [15] This insight implies that there is a significant learning potential in knowledge transfer from a successful scientific field, for example, systems science, to one that is still searching for answers, namely, U.S. health care. The evolution of U.S. health care has not followed any known organizational principles, and certainly not systems science. The unfavorable outcome of such an expansion can now be corrected by the transposition of systems science knowledge onto the restructuring plans for U.S. health care.

Boulding conceptualized systems' interrelationships by stating that "all lower level systems are embedded in systems of a higher order [and become their sub-systems]...but higher level systems have unique characteristics." [12] This means that a lower system's characteristics are represented in a higher system but that the higher system expresses its own specific features. Complex adaptive systems retain, on balance, a lower system's characteristics that are considered to enhance the inter- and intrasystem functioning of a larger system. Simultaneous discarding of counterproductive/nonfunctional attributes from a system, however, must also be present as a proactive process in a system's ongoing evolutionary adaptation; otherwise, the accumulation of nonfunctionality would lead to higher entropy. Following these guidelines would greatly improve health care.

Ashby's theorem of requisite variety addresses the issue of external control over a system. [11] It teaches that in order to achieve successful control of a system's variables, all variables have to be under our control. Attempting to control only some variables, even if they are significant, may destabilize a system rather than facilitate its improvement. Here lies the danger of unilaterally attempting to control health care through individual variables such as costs, physicians' services, medications, and so on. Two main issues are interacting within this concept: *variety*, which reflects complexity, and *control *of a system. An open system cannot be unilaterally controlled unless it is converted into a closed system. An increasing complexity of a system without a corresponding expansion of reciprocity of horizontal and vertical hierarchy, up to a proportionate and system-specific boundary, will likely encounter turmoil and dysfunction. (Additional file 1, Part 6)

**Figure 1:**
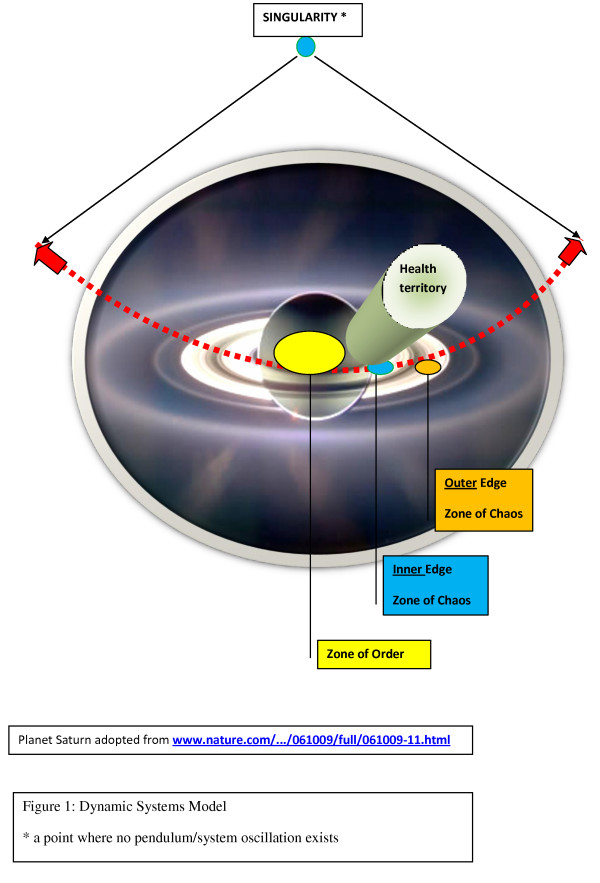
**Dynamic Systems Model**.

**Figure 2:**
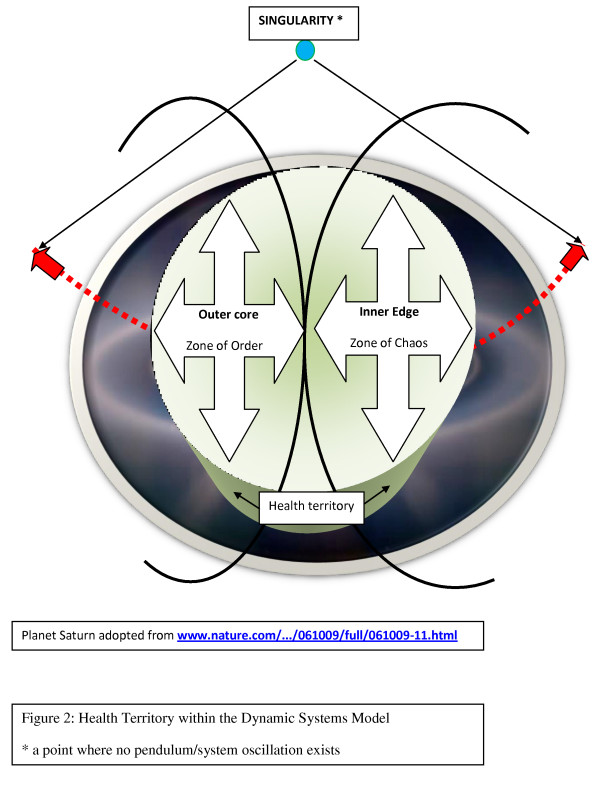
**Health Territory within the Dynamic Systems Model**.

**Figure 3:**
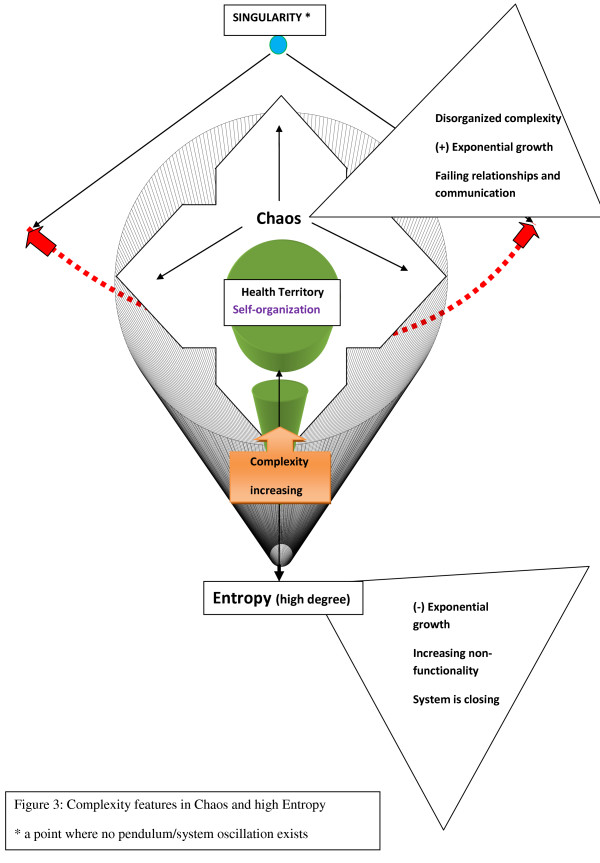
**Complexity features in Chaos and high Entropy**.

A dynamic systems model of normal and abnormal oscillations of biologic entities such as cells or organizations has been developed. It incorporates systems science, complexity, and chaos theories. [7] This model conceptualizes the existence of zones of order, chaos, and a high degree of entropy with ongoing, plausible pendulum-like transitions, from the initial state to the end state following many physiologic resetting. (Additional file 1, Part 10) The center of this model lies in the health territory, that is, a steady state of a system's dynamic balance that straddles the outer core of the zone of order and the inner edge of chaos. The health territory is an active space of self-organization and self-adaptation within a nonlinear dynamical system following the principles of organized complexity; stable frontier depends on the efficacy of resetting mechanisms. Outside of the frontier of a well-functioning system's health territory, any biologic entity/organization can enter either further into the zone of chaos or a zone of a high degree of entropy, each dramatically affecting a system. (Fig. 1, 2, 3)

A fully functioning open system expresses a number of defining characteristics. Among the predominant features are relationships among the components utilizing the same information transfer as well as self-organization and self-adaptation within a defined semipermeable boundary that have the potential to create a new emergence. A system's stability depends on the quality and quantity of patterned relationships and the corresponding hierarchies. In an open system, the balance is not just a reflection of its internal relationships (self-organization) but also its larger external relationships to larger systems (self-adaptation). A system's resiliency is related to its redundancy. (Additional file 1, Parts 4, 7 and 12)

Most difficulties of a large system can be traced to failing smaller systems known as subsystems. A deteriorating subsystem that has the potential to threaten its larger system has escaped the system's self-organizing controls; such a development places this system at the outer edge of the zone of chaos.

The inner edge of chaos has functional characteristics that can be plotted as an initial segment of an exponential curve that runs in near proximity to a straight line; such a schema represents a normal rhythm. At a certain point on the exponential curve, however, there is a significant divergence between the changes expressed by a straight line and the exponential ones, an inflection point signifying a dramatic change of slope; the outer edge of chaos begins here. The important ingredients of a functioning system, relationships, communications/information transfers, and organized complexity are breaking down along this sloping path. The structural and functional dilemmas of being in the zone of chaos have to be eventually resolved by the biologic entities or organizations, either by returning to physiologic oscillations or by allowing the disorganized complexity to eventually overcome a system. [16]

The final phase of a pendulum-like oscillating system in this dynamic model is the devolutionary phase of a system. It represents the opposite of the initially traveled evolutionary stage, and it expresses biologic symptoms with organizational correlates, such as stagnation, degeneration, inflammation, and senescence. This phase also is located outside of the health territory and precedes the end state. During this phase, a system loses its organized complexity, self-organization, and self-adaptation, and it looks less and less like an open system. Randomness and disorganized complexity have returned, and functionality is decreasing because of the prevailing high degree of entropy; the whole system is closing down, with a minimum of energy and information exchange.

Physiologic resetting is the guardian process of the health territory allowing oscillations within its boundaries. It is defined as a point of active interference with a given cycle trajectory as it approaches its climax of criticality. Either resetting of an old cycle around previous singularity, a point where no pendulum/system oscillation exists, takes place, or a new cycle, based at a singularity of differentiation, begins. Resetting also is a stimulus that can propel a biologic entity or an organization from one zone to another. In its simple form, resetting may be considered a switch that can, however, turn into autocatalysis of either only positive or only negative feedback loop reinforcements. The resetting impetus can be either internal and/or external.

Complexity is the governing pattern of interactions within a system. It expresses the functionality of a system and reflects the process of relationships that produces self-organization and self-adaptation. Complexity begins to appear at a distance from the initial state, when randomness gives way to self-organization. It is the "spontaneous emergence of order... [allowed by] a constant flow of energy and matter through the system... [which produces an] emergence of new structures and new forms of behavior... [which are] the hallmark of self-organization. [It] occurs only when the system is far from equilibrium." [13] Self-organization reflects the evolving interactions of innumerable relationships of a system's components.

Complex adaptive systems (CAS) express a high degree of resiliency and robustness to environmental challenges through their self-adaptation and internal self-organization. "One important characteristic of CAS is that...the control of a complex adaptive system tends to be highly dispersed." [17]

The outer core of the zone of order does engage in some functional adaptation; a system is responsive to changes within a certain range. When the outer core combines with the inner edge of chaos, it forms the health territory, where a system's components are routinely replaced but the basic relationships and methods of communication/information transfer persist. In this healthy state, living entities or organizations are undergoing renewals, the self-controls are functioning well, and innovation flourishes.

The outer edge of chaos represents a far-from-equilibrium state where self-controls are not functioning and innovation is distorted. The components have stopped communicating, breaking off system-wide relationships and competitively maximizing their own growth at the expense of a larger system; some feedback loops still persist, but only to facilitate the siphoning of energy from this system. From here, biologic entities or organizations may either undergo the final exponential uncontrolled growth or enter the path of self-destruction.

**Figure 4:**
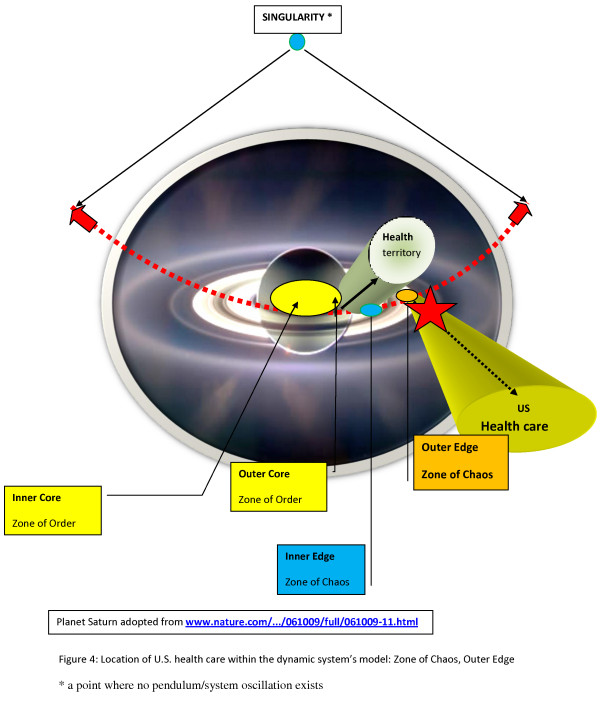
**Location of the U.S. health care within the Dynamic System's Model: Zone of Chaos, outer edge**.

**Figure 5:**
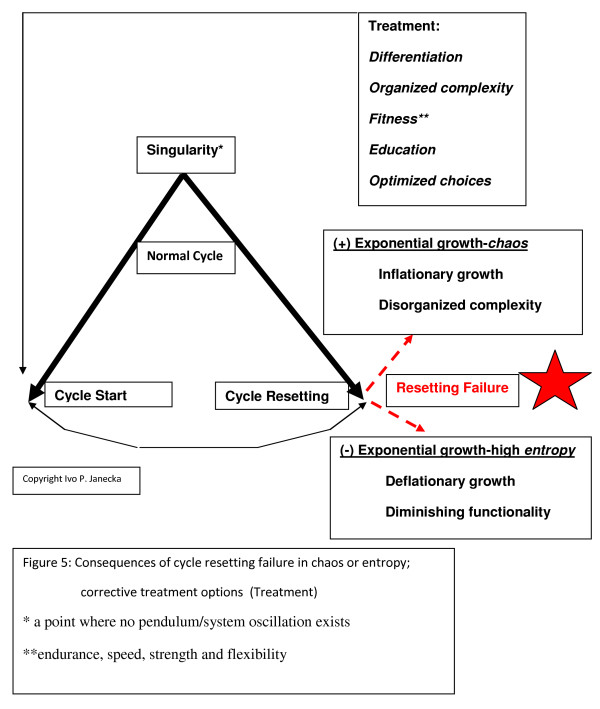
**Consequences of cycle resetting failure in chaos or high entropy; corrective treatment options**.

This comparative analysis of U.S. health care to a complex adaptive system, which was based on two research questions (i.e., Is the U.S. health care a system? and What is the state of its functioning?), led to the following observations:

A comparative analysis of a key system's characteristics and U.S. health care revealed that U.S. health care is an incomplete system. Its operations take place at the outer edge of chaos, far removed from the optimal zone of functioning, which is the health territory, as delineated in the dynamic systems model; solutions, however, did emerge. (Additional file 1) (Fig. 4, 5)

U.S. health care is not an all-inclusive organization; millions are lacking access and active participation. For health care to comply with a key system's principle to encompass numerous and variable components, it needs to include all entities in the United States. By many measures, health care also is not creating consistent value among its member components; value in this context is a synonym for a system's ultimate goal: its emergence. Such value or a system's emergence in health care can be expressed as an equation where quality, defined as efficient, effective, and risk-averse service, is a numerator, divided by cost. The viability of this equation requires that all system components be actively engaged in system optimization, from individual responsibility for *health *to societal responsibility for *care*. On all societal levels, health has to be incentivized, not care. (Additional file 1, Parts 1 and 5)

Many relationships among healthcare components, such as for-profit HMOs, focus primarily on maximizing their activities regardless of the overall impact on a system. Such an approach compromises the relationship dynamics of an entire system, which need to focus principally on optimizing a system through well-balanced feedback loops.

Health care as an organization is better characterized as being complicated than complex because it reflects disorganized complexity, a characteristic of the zone of chaos in the presented dynamic systems model. (Fig. 1, 2, 3). Reestablishing organized complexity would be an expression of the functionality of a balanced system. (Additional file 1, Part 2)

Communication within health care and with its external environment is greatly handicapped by existing gaps in health literacy and depositories of data, information, and knowledge. These discrepancies contribute to unhealthy choices on all societal levels, including individual lifestyles and policy decisions. Interoperability on all planes of communication, epitomizing a uniform information transfer, is the backbone of an optimal system's relationships, which resemble a self-learning neuro-net. The lifelong education of all system components is a prerequisite. (Additional file 1, Part 3)

There has been a chronic disconnect in health care between the horizontal and vertical hierarchies (patients/providers vs. policymakers). A system's capacity for adaptation is best served by unhindered and always-evolving reciprocity between these two hierarchies.

The current healthcare boundary is not semipermeable; it is either too rigid (e.g., the existence of insurance represents a major barrier to entry) or too loose (e.g., a legislative mandate without fiscal support that instructs every emergency room to accept all patients, including the uninsured, causing great network dislocation). The boundary participates actively in a system's self-adaptation to the external environment while protecting its structural integrity and defining a system's optimal neighborhood of functioning.

The external environment for health care is part of a larger societal system; both influence and adapt to each other through boundaries. A system's balance is strongly influenced, positively or negatively, by ongoing interactions. For example, the high cost of medical supplies/equipment (e.g. pharmaceuticals, scans, etc.) is derived from the manufacturers' focus on maximizing their own domain instead of contributing to the overall system optimization of which they are members. As a consequence, the self-adaptation of the affected system is damaged. A step in the right direction, in this regard, would be impartial regulation seeking optimal functioning of the affected system.

Within health care, many smaller systems are out of synch. Systems science requires the interrelatedness of smaller and larger systems expressing self-affinity and self-similarity, as well as cycle congruity. (Additional file 1, Part 8) Synchronizing, for example, the larger ecologic and climate systems/cycles is a prerequisite for the long-term sustainability of health care. This is because these cycles have dominant influence on all systems with a biologic underpinning. Disease occurrence or progression may be related to the Earth's latitude or the incongruence with day/night cycles. The interrelatedness of systems reflects the potential ripple effect of all choices made, regardless of the initial scale and location; if a system is already in the zone of chaos, these influences can be significantly magnified. Choices are understood as actions taken following the amalgamation of information filtered through our senses, cognition, memory, information transfers, and emotions; ultimately, they determine our interaction with the environment. A good example is the strong relationship of socioeconomic status (i.e., the reflection of education, skills, earnings, etc.) to health. It does not seem to be the absolute wealth level that has health consequences; rather, it seems to be the rank that specific wealth provides within the community.

An open system's fitness (i.e., endurance, speed, strength, and flexibility) is an outcome of an ongoing balance/steady state among its energy input, throughput/internal metabolism, and energy output. Excessive intake and limited output negatively impact metabolism and result in metabolic/organizational instability. The consequences of this lopsided energy gradient are applicable not just to a single human body, where this disequilibrium results in obesity and associated problems, but also to society as a whole, with similar but larger scale consequences. (Additional file 1, Part 4).

A system's own cycle needs to be in synch with larger cycles. A system will have a vastly different response to its own or environmental disequilibrium, depending upon where it is within the dynamic model's trajectory. As a consequence, corrective measures also will lead to a system's variable response due to the functional prevalence of organized versus disorganized complexity, the dominance of chaos, or high entropy principles. (Additional file 1, Part 2)

## Discussion

A basic matrix of any nation can be seen as a large societal system stratified into multiple, smaller systems/subsystems, including health care, where each person represents an essential component. According to systems science, any system, large or small, can be classified either as a true system, implying full compatibility with systems science principles, or an incomplete system. The dynamic systems model then allows a space-time functional positioning of a system that broadly reflects its health or disease status; it also offers principle pathways to recovery if a system is considered outside of the favorable health territory.

Health care, designed to reestablish the health of a large societal system, must seamlessly incorporate all of its subsystems arranged in ever-expanding circles of influence. They may be schematically named "self health/care," "family health/care," village health/care," and so on, terms indicating categories of responsibility for health as well as care.

The human body is the best known system. Its metaphor can be used to not only diagnose any organization as a functional or a dysfunctional system but also prospectively evaluate any intended intervention as system optimizing or system destabilizing. A series of questions, with a reference to any proposed new healthcare intervention, could be as follows: Is the proposition system compliant? Is there a biologic analogy to the plan? For instance, should all societal members be included in the healthcare system? The answer, through the biologic comparative metaphor of the human body as the best system, would be a resounding "yes" because all bodily components are fully integrated into its whole system. It would seem preposterous to accept the fact that some units/cells/organs could be intentionally excluded within a well-functioning system. Hatch in her book, *Organization Theory*, emphasizes that "...metaphor...[represents a] useful means of recognizing and understanding the essence of a given phenomenon." [14] It allows you " [to] understand one kind of experience in term of another by suggesting an identity between two things that you would not normally consider to be equivalent...So long as you understand one element of the metaphor, you can learn something about the other...metaphor encourages you to explore the parallels between an object of interest and something that is...known to you" [16] The general systems theory puts a scientific base under this observation that it terms isomorphism.

It may be argued that the use of a biological system to gain a comparative understanding of a social system might be precluded, for example, by the presence of intention and knowledge in the highest biologic system, the human body. The congruence of all biologic systems, however, lies in their basic uniformity of function and structure when looked at through the framework of systems. They are all open systems with intake, throughput, and output, all of which attempt to maintain homeostasis within the external environment. They maintain a viable boundary that allows intersystems exchanges. The capacity for intent has been traditionally seen as only human mental function rather than a complex process within interconnected physical matrix. Most known mental functions do seem to have physical substrate resonance within the central nervous system and its connections. Our thoughts, which can be seen as activation of the frontal cortex, are often preceded by hypothalamic activity which provides an emotion-activated filter to our final thought product. From this point of view, mental and physical processes, in relationship to our thoughts, seem to have common activation pathways. As the debate continues, what is often referred to as a mental state actually reflects the amalgamation of genetic predispositions, epigenetic/cultural influences, sensory inputs, and individual's operational paradigms processed through emotional filters. Health care, as a system, can be seen as pooled individuals, including consumers, providers, payers, legislators, etc., who are all subject to this complex process of intent creation and knowledge integration within their limited and delimited boundaries. Considering this framework could allow a comparison of a biologic system, the human body, with the existing system of health care. As a consequence, the massive impact of the behavioral and lifestyle choices on all major diseases and all-cause mortality can be viewed in more focused relevance to the existing body of knowledge regarding biology, organizations, and systems. [17]

All existing knowledge, including the need for differentiating quantifiable risk from uncertainty, is imperfect and continues to evolve; some changes are continuous, while others occur in various leaps of timeframe and depth of understanding. Some knowledge, however, has been reasonably constant for extended periods of human existence even though, the science behind it has changed. For example, to live in accordance with day/night and seasonal cycles has been intuitively and empirically felt by people to be positive, likely starting from the early days of our existence. But, it was not until relatively recently that science has documented the health benefits of living congruently with these cycles as well as pointing to the negative impact of living within prolonged cycle incongruence. Science has also elucidated biologic changes of similar circadian and seasonal oscillations and their relationships to health within the animal kingdom giving further support to the feasibility of biologic and social systems comparison.

In a process of comparative learning on a large and diverse scale, as this study attempted, in order to gain insight into health care from a theoretical model, systems science, and the knowledge of human body, it is not possible to have a perfect match among these reference points. This fact, however, should not mitigate the value of the potential constructive isomorphic knowledge that can be gained from such an approach. Clancy, for example, already looked at hospitals as complex systems that can benefit from systems science in the areas of planning and management. [18] Many others have recently contributed to this field. For example, Schwaninger felt that coping with complexity through rationality is at the heart of the systems approach. [19] Rouwetter et al. explored the factors that influence rationality, including strength of feedback, exogenous change, decision interval, model transparency, decision information, decision strategy, mental model/cognitive style, and number of players. [20] Wittmannn and Hattrup identified a relationship between intelligence and rationality of a decision process in a real world full of nonlinearities. [21]

The fundamental goals of functional and sustainable health care lie in creating it as an open, complex, and adaptive system that is fully compliant with systems science principles; many existing deficiencies have already been identified. [22-33]. Such a system is composed of many and variable components to assure its evolutionary progression. All components need to be engaged in relationships of organized complexity, which arises as a consequence of self-organization facilitated by feedback loops utilizing common information transfer. The system-surrounding, semipermeable boundary simultaneously protects the encompassed system and assists in its internal and external adaptation to its environments because of its selective functionality. (Additional file 1, Part 1)

This comparative study of U.S. health care with a model of a complex, adaptive system demonstrates that currently fragmented health care is a system in the process of failing, exhibiting signs of disorganized complexity, chaos, and high entropy, all representing detrimental states. The implications of this study highlight the need for U.S. health care to become fully compliant with systems principles as the only long-term, viable solution to the existing healthcare predicament. To start forming a foundation, however, is an immediate need. Any changes we make today and tomorrow need to fit into the blueprint of systems science-based health care.

## Concluding Statement

Systems science provides a unique template of organizational principles because it is supported by biologic findings. All organizational entities can be compared to those principles; any incongruence indicates an incomplete or an already failing system. Only a fundamental restructuring along systems science principles can ensure the robustness and resiliency of a new organizational system.

The described comparative study of U.S. health care and the systems science principles revealed that U.S. health care is an incomplete system in the zone of chaos charted by the dynamic systems model. The implications, which are supported by chaos theory, are that anything less than a fundamental intervention may further destabilize the current system in the process of failing, with additional high probability that even a small future change could bring calamitous consequences. By complying with systems science principles and the congruence of pertinent cycles, U.S. health care could dramatically improve its value creation for the whole of society as well as its resiliency and long-term sustainability.

The following immediate steps could be taken:

• Prioritize self-health and self-care because the "self" represents the smallest subsystem and needs to be recognized as the essential/starting component of the larger healthcare/societal system.

• Incentivize health, not care.

• Take immediate steps toward restoring a positive energy/fiscal gradient within the entire system by introducing a combined life and health insurance for all individuals, thus assuring a system of positive financial gain after a period of negative loss. Establish system-wide functional reciprocity between horizontal and vertical hierarchies.

The principles for the creation of a systems science-congruent healthcare system are known. The guide posts exist, but the journey is up to us.

## Supplementary Material

Additional file 1 Relationship of systems science principles and US health care. Comparison of features between a complex system, mirrored in the human body, and health care including corrective solutions. Click here for file
