# Supplementary material for: Is U.S. health care an appropriate system? A strategic perspective from systems science
Source: Health Res Policy Syst. 2009 Jan 2;7:1. doi: 10.1186/1478-4505-7-1 (PMC2629469; doi:10.1186/1478-4505-7-1)
Supplement: Additional file 1 — Relationship of systems science principles and US health care. Comparison of features between a complex system, mirrored in the human body, and health care including corrective solutions. [file 1478-4505-7-1-S1.pdf]

COMPLEX SYSTEM / HUMAN BODY	HEALTH CARE	SOLUTIONS
1.Numerous & variable components which generate system's emergence/value; all components participate	Not all are included; value is inconsistent; unfocused responsibility [22-24]	All must be included; greater value to be created; responsibility for health-from individual to society
2.Relationships optimize system with feedback loops	Relationships are not pro-system	Integrate all relationships
Organized complexity characterizes function	Disorganized complexity [23-25]	Reestablish organized complexity
3.Communication is reciprocal and resembles a self-learning neuro-net	Communication is non-uniform; choices are not based on existing knowledge [23,26-30]	Interoperability of communication; life-long education
4. System seeks homeostasis/steady state	Choices override system needs	Set energy balance via diet/exercise/stress management; track fitness/performance status;* increase epigenetic research
5.Balance of function/dysfunction leads to cost/energy stability	Health/disease imbalance increases cost/energy loss [23,26-30]	Combine life and health insurance
6.Horizontal and vertical hierarchies are reciprocal	Vertical hierarchy controls [24,31]	Reestablish reciprocity; create system-optimizing policies/tort/ research support
7.Boundary is semipermeable; in synch with external environment	Boundary is defective; poor adaptation to environment	Reestablish semipermeability; flexible adaptation**
8.All-systems relatedness exists	Many systems don't relate [26]	Connect all systems
9.Systems change with cycles	Diffuse asynchrony [32]	Synchronize system with other systems/cycles (ecologic, climate)
10.Systems' cycles are intelligently reset	Resetting through crises [6]	Introduce rational resetting
11.Systems are of proportionate size to maintain viability of feedback loops, semipermeability of boundary, and actionable resetting	Disproportionality exists [30]	Allow re-sizing of healthcare units/subsystems through self-organization
12.Systems show balanced growth as outcome of reciprocal hierarchies	Imbalanced growth [33]	Formulate mission/goals/ authority/functional space for each hierarchy

Additional file 1: Comparison of features between a Complex System, mirrored in the Human Body, and Health care with corrective Solutions. (*endurance, speed, strength and flexibility) (**impartial regulation seeking optimal functioning of the system)
